# Neoatherosclerosis after Drug-Eluting Stent Implantation: Roles and Mechanisms

**DOI:** 10.1155/2016/5924234

**Published:** 2016-06-30

**Authors:** Yuanyuan Cui, Yue Liu, Fuhai Zhao, Dazhuo Shi, Keji Chen

**Affiliations:** ^1^Cardiovascular Diseases Center, Xiyuan Hospital, China Academy of Chinese Medical Sciences, Beijing 100091, China; ^2^China Heart Institute of Chinese Medicine, China Academy of Chinese Medical Sciences, Beijing 100091, China

## Abstract

In-stent neoatherosclerosis (NA), characterized by a relatively thin fibrous cap and large volume of yellow-lipid accumulation after drug-eluting stents (DES) implantation, has attracted much attention owing to its close relationship with late complications, such as revascularization and late stent thrombosis (ST). Accumulating evidence has demonstrated that more than one-third of patients with first-generation DES present with NA. Even in the advent of second-generation DES, NA still occurs. It is indicated that endothelial dysfunction induced by DES plays a critical role in neoatherosclerotic development. Upregulation of reactive oxygen species (ROS) induced by DES implantation significantly affects endothelial cells healing and functioning, therefore rendering NA formation. In light of the role of ROS in suppression of endothelial healing, combining antioxidant therapies with stenting technology may facilitate reestablishing a functioning endothelium to improve clinical outcome for patients with stenting.

## 1. Introduction

The emergence of first-generation drug-eluting stents (DES) has greatly minimized the limitations of bare metal stents (BMS); however, concern about in-stent neoatherosclerosis (NA) has attracted much attention owing to its close association with late complications such as revascularization and late stent thrombosis [1–3]. NA is characterized by accumulation of yellow-lipid-laden foamy macrophages within neointima with or without necrotic core and/or calcification after stent implantation [4]. Pathological studies have revealed that advanced NA with neointimal rupture and thrombosis is the most common mechanism of definite vary late stent thrombosis and is also associated with a high frequency of ST-segment elevation myocardial infarction [5, 6].

Theoretically, accomplishing endothelial coverage after stenting is considered to be safe to prevent against vascular pathological changes, including thrombosis and inflammation. Nevertheless, “neointima” of DES seems not effective as normal because recent studies have reported that patients with first- and second-generation DES have a high frequency of NA, which was less seen in bare metal stents (BMS) [7, 8]. DES-induced endothelial dysfunction plays a critical role in NA formation [9, 10]. Here, we try to describe the detailed pathological and cellular mechanisms for DES-induced NA.

## 2. Clinical Evidence of Neoatherosclerosis

Traditionally, promoting stent coverage after stenting is considered as an important index to evaluate efficacy and safety of current or advanced stents. However, neointimal function after stenting seems anxious for the emergency of NA. Data from the CVPath (Gaithersburg, Maryland) stent registry, including 209 first-generation DES (103 sirolimus-DES [SES] and 106 paclitaxel-DES [PES]) and 197 BMS with implant duration of >30 days, showed that the incidence of NA was greater in DES (30%) than in BMS (16%; *P* < 0.001) [1]. Furthermore, Ali et al. [7] analyzed 65 symptomatic patients with in-stent restenosis (ISR) 33 months of follow-up, finding that optical coherence tomography- (OCT-) verified NA was greater in first-generation DES than with BMS (68% versus 36%, *P* = 0.02). These results suggest that NA is more prevalent in first-generation DES when compared with BMS.

The advent of second-generation DES, including everolimus-eluting stents (EES) and zotarolimus-eluting stents (ZES), has been improved with more biocompatible polymers and thinner strut stent backbones than those with first-generation DES [11]. Neointimal growths developed within those second-generation DES have been documented. Kim et al. [12] reported that second-generation DES (ZES; Endeavor) facilitated strut coverage and reduced rate of malapposed strut compared with first-generation DES. In contrast, results from another multicenter OCT analysis, including 212 patients with first- or second-generation DES (second-generation DES: 40 zotarolimus, 36 everolimus, and 35 biolimus; first-generation DES: 65 sirolimus and 36 paclitaxel), showed that the second-generation DES was not more protective against NA than the first-generation DES [13]. Even second-generation cobalt-chromium EES (CoCr-EES), possessing greater strut coverage with lower incidence of late/very late stent thrombosis than SES and PES in human autopsy analysis, findings showed that the frequency of NA in CoCr-EES was not statistically different when compared with first-generation devices [14]. These results indicate that “neointima” of DES is not functional as normal vessels, and further examination of the neointimal components of DES is needed.

## 3. Pathological Characteristics of Neointima

Neointima of DES has several features distinguishing from these of BMS, including its components, developing time, progression, and prognosis. Pathological studies, including coronary angioscopy, intravascular ultrasonography (IVUS), histological analysis, and OCT researches, have been performed to investigate the features of neointima in both DES and BMS-associated segments, as shown in Table 1.

Early histopathologic studies revealed that neointimal components of DES lesion were similar to those in BMS where the neointima was mainly composed of proliferative smooth muscle cells with proteoglycans-rich extracellular matrix. However, emerging evidence suggests that neointimal components of DES are of obvious difference compared to BMS. Hara et al. [15] conducted a rabbit study by using intravascular near-infrared fluorescence (NIRF) molecular imaging (high-resolution imaging of fibrin) in combination with simultaneous OCT, showing greater fibrin deposition and fibrin persistence in DES than in BMS at 7 and 28 days, respectively. Moreover, neointimal tissue of DES (*n* = 34) and BMS (*n* = 27), identified in 61 lesions by using iMap IVUS which allows identifying neointimal tissue components in vivo, showed that the neointima in DES placement showed smaller fibrotic component (67% versus 78%, *P* < 0.0001), larger necrotic (14% versus 9%, *P* < 0.0001), and calcified (15% versus 7%, *P* < 0.0001) components compared to BMS [16]. These results suggest that fibrin deposition rather than endothelial cells is a major surface component for DES-neointima [17], indicating that neointima with less endothelial cells in DES does not possess its native functions.

Lipid deposition within neointima of DES is one of the most characteristics which differs from BMS, where the latter is mainly composed of smooth muscle cells. Nakano et al. [18] examined the neointimal characteristics of both DES and BMS and demonstrated that neointimal compositions of DES restenosis showed greater proteoglycan deposition and less smooth muscle cellularity over time, whereas BMS showed greater cell density and collagen deposition. Furthermore, Ali et al. [7] reported the findings using OCT and NIFS with IVUS for patients with ISR, finding the total lipid core burden index and the density of lipid core burden index (34 versus 9, *P* < 0.001; 144 versus 26, *P* < 0.001, resp.) to be higher in DES than in BMS. Similarly, Yonetsu et al. [19] revealed a greater incidence of lipid-laden plaque (37% versus 8%, *P* = 0.02) and a higher percentage of lipid-rich plaque (12.9% versus 1.2%, *P* = 0.01) that were found in DES compared to BMS within 9 months. Interestingly, a significant difference in the regression of NA is notable in DES and BMS. Awata et al. [20] reported a serial angioscopic evidence of neointima after SES and BMS up to 2-year follow-up, revealing that yellow plaques were exposed in 71% of SES at the first follow-up (3.6 ± 1.1 months) and remained exposed until the third follow-up (21.1 ± 2.2 months), whereas yellow plaque in BMS has disappeared by the time of the second follow-up (10.5 ± 1.6 months). Of note, to assess neointimal hyperplasia after DES placement, data from a clinical study including 37 angina patients undergoing repeated percutaneous coronary intervention showed that the late phase (mean follow-up, 40 ± 23.9 months) had a greater percentage of lipid components and relative larger necrotic volume compared to the early phase (<1 year), suggesting that lipid plaque of DES grew persistently and rapidly with time [21]. Lipid deposition within neointima in DES segments seems to increase risks of adverse events. On the one hand, the prevalence of thrombus was significantly higher on the yellow than on the white neointimal area [22]. On the other hand, patients with yellow plaque after stenting showed great frequency of late stent failure, including cardiac death, acute myocardial infarction or unstable angina, or need for revascularization associated with stent site, compared to those without yellow plaque (8.1% versus 1.6%, Log rank *P* = 0.02) [23].

Clinical reports and pathological findings from patients with DES postulate a hypersensitivity inflammation as an important factor in neoatherosclerotic development [23, 24]. Potential culprits responsible for hypersensitivity include arachidonic acid metabolites, proteolytic enzymes, and inflammatory cells such as macrophages, T-lymphocytes, mast cells, and eosinophils [25]. Pfoch et al. [24] reported that patients with a polymer-based PES (Taxus, Boston Scientific) presented with characteristics of hypersensitivity: disseminated wheals, pruritus, bronchial asthma, and synovitis and were cured by initial intravenous injection followed by oral antihistamine treatment for 1 month until paclitaxel was eluted totally. Moreover, in noninjured coronary arteries of domestic swine (*n* = 58), overlapping stents (SES, PES, and BMS) were implanted to detect circumferential granulomatous inflammation, defined as inflammation consisting of macrophages, multinucleated giant cells, lymphocytes, and granulocytes, including many eosinophils in stented segments. Results showed that circumferential granulomatous inflammation was more prevalent in SES (9 of 23, 39%) compared with BMS (0 of 44) in the combined 90- and 180-day cohort. Recently, Niccoli et al. [26] considered that mammalian target of rapamycin- (mTOR-) inhibited DES stent type is associated with an increase of eosinophil cationic protein in serum levels of patients undergoing stent implantation, possibly triggered by permanent polymer. However, Fu et al. [27] analyzed neointimal coverage of polymer-free sirolimus-DES in coronary arteries of 8 normal swine; lymphocyte infiltration of peristrut was more frequently seen in heterogeneous sections than in homogeneous sections. And Cook et al. [28] reported histology findings in 54 patients with DES (28 patients with very late DES stent thrombosis and 26 controls), suggesting that very late DES thrombosis was associated with histopathological signs of inflammation, and eosinophilic infiltrates were more common in thrombi harvested from very late DES thrombosis. These results suggested that antiproliferative drugs-mediated DES may play a role in hypersensitivity, partly contributory to promote progression of NA.

NA is classified as I (thin-cap NA), II (thick-cap NA), and III (peristrut NA) types, topographically. The types of NA between DES and BMS are discrepant. Ali et al. [7] detected the cap neointima of both DES (*n* = 51) and BMS (*n* = 14) in 65 patients with in-stent restenosis (ISR) at 33-month follow-up and suggested that the type 1 thin-cap neoatheroma was greater in DES than BMS (20% versus 3%, *P* = 0.01). Similarly, Ando et al. [29] also found this phenomenon in patients with ISR after SES (*n* = 20) or BMS (*n* = 34) implantation, finding that a smaller fibrous tissue percentage in neointimal tissue by integrated backscatter IVUS was observed in SES than in BMS (72.6% versus 82.0%, *P* = 0.011). Moreover, Kang et al. [30] analyzed 50 patients (30 stable, 20 unstable angina) with 50 DES-ISR with 32.2-month follow-up, revealing that 52% of lesions had at least 1 OCT-defined in-stent thin-cap fibroatheroma- (TCFA-) containing neointima, and patients presenting with unstable angina showed a thinner fibrous cap (0.55 mm) and higher incidence of OCT-defined TCFA-containing neointima compared to stable patients. Like vulnerable plaque characterized by a thin cap of lesions (<0.65 mm), these results suggest that NA of DES is responsible for late complications for its favor of rupturing over time.

## 4. Cellular Mechanisms of Neoatherosclerosis

Many pathological factors have been elucidated as indicators of NA, such as delayed arterial healing [12], hypersensitivity [26], stent types, stent age, and patient characteristics [31, 32], including current smoking and chronic kidney disease. Antiproliferative drugs-induced incomplete reendothelialization plays a key role in neoatherosclerotic development. Nakazawa et al. [33] analyzed 44 New Zealand White rabbits with induced atheroma by bilateral iliac artery stents to evaluate endothelial coverage between DES (SES, ZES, and EES) and BMS. Findings showed a significant lower level of endothelial nitric oxide synthase (eNOS) expression in DES than BMS, although endothelial coverage was comparable between DES and BMS, suggesting that regenerated endothelium was dysfunctional in DES. Therefore, antiproliferative drugs such as sirolimus and paclitaxel inhibit endothelial cells proliferation and induce endothelial dysfunction which contributes to NA.

### 4.1. Reactive Oxygen Species (ROS) Production Induced by Antiproliferative Drugs

Jabs et al. [8] analyzed the effect of sirolimus in vascular dysfunction using Wistar rats undergoing infusion (5 mg/kg/day) for 7 days for mimicking the continuous sirolimus exposure of a stent vessel. Results showed that sirolimus caused a marked endothelial dysfunction and a desensitization of the vasculature to the endothelium-independent vasodilator nitroglycerin; moreover, upregulated superoxide production was observed, in part by nicotinamide adenine dinucleotide phosphate (NADPH) oxidase via stimulating of p67phox/rac1 expression and increased rac1 membrane association. Similarly, paclitaxel also increases ROS production via activation of NADPH oxidase, including p47 (phox) mRNA and gp91 (phox) mRNA in arteries and human coronary artery endothelial cells, while the level of nitric oxide (NO) is reduced [34]. In view of the ROS production, evidence suggests that ROS may mediate endothelial caveolae-mediated transcytosis [35] and paracellular pathway [36] which are potentially associated with neoatherosclerosis development. Detailed explanations will be discussed in the following sections.

Transcytosis of vascular endothelial cells is a basic process for maintaining vascular homeostasis [6]. Caveolae-mediated transcytosis is a major conduit for transporting macromolecules (>3 nm of molecular radius), including albumin, insulin, and LDL, from one side of a cell to the other via membrane-bounded caveolae [37–39]. Known that many excellent articles about caveolae structure and functions have been introduced in detail [40, 41], our focus is on the effects of ROS on neoatherosclerotic development via caveolae-mediated transcytosis pathway.

### 4.2. ROS Promote Lipid Uptake via Caveolae-Mediated Transcytosis

Impairment of endothelial barrier function is implicated in many vascular disorders. One prevalent mechanism of endothelial dysfunction is an increase in ROS under oxidative stress. As mentioned in pathological studies, both sirolimus and paclitaxel-eluting stents are associated with increased vascular level of ROS, consequently altering endothelial function in the treated artery [42]. Although there is no direct evidence to demonstrate the effects of DES in increasing lipid uptake, ROS-associated lipid deposition in vessels is widely described. By exposing an aortic endothelial smooth muscle cell bilayer to a free-radical generating system to detect the influence of superoxides in lipid permeability and uptake, increased level of ^125^I-LDL uptake was observed via transcellular pathway [43]. Moreover, transcytosis of FITC-labelled LDL simulated by C-reactive protein (CRP) was examined in human umbilical vein endothelial cells and ApoE(−/−) mice, pointing out that CPR-stimulated LDL uptake within in vitro and in vivo was increased closely associating with increased ROS production. This action was blocked by an NADPH oxidase inhibitor (inhibition of ROS production) [44]. Therefore, these results indicate that ROS production stimulate lipoprotein uptake in endothelial cells.

ABC transporters (such as ABCA1 and ABCG1) play an important role in cholesterol homeostasis, especially in liver. In contrast to smooth muscle cells and macrophages that form the atherosclerotic plaque, neither ABC transporters nor scavenger receptor B-I is required for vascular endothelial cholesterol efflux, indicating that other pathways may be involved in the lipid transport for endothelial cells [45]. Caveolae-related transcytosis and their generated channels are the main cellular instruments for trafficking cargoes in endothelial cells. Endothelial-specific cav-1 expression is essential for the progression of atherosclerosis [46]. cav-1, as a primary structural protein of caveolae, greatly regulates caveolae formation and caveolae-mediated endocytosis and transcytosis in microvascular endothelial cells [47]. Many studies have already investigated the relationship between ROS and cav-1-mediated caveolae pathway [48, 49].

Evidence showed that ROS upregulate cav-1 protein expression via increasing binding of Sp1 to the cav-1 promoter region containing the two GC-rich boxes [49]. Overexpression of cav-1 inhibits thioredoxin reductase-1 (TrxR1), an important antioxidant enzyme that controls cellular redox homeostasis. Lack of TrxR1, which fails to localize to caveolae and binds to cav-1, constitutively induces oxidative stress-mediated endothelial dysfunction [50]. Some key signaling pathways, including activation of* Src* kinase family members and extracellular signal-regulation kinases (ERK1/2), have been specifically incriminated in ROS-mediated barrier disruption.* Src* signaling has been demonstrated to be a crucial “switch” in the regulation of caveolae-mediated transcellular transport via phosphorylation of cav-1 at tyrosine 14, followed by phosphorylation of dynamin-2 to fission of caveolae. P90 ribosomal S6 (p90RSK) is a potentially important downstream effector of* Src* and ERK1/2. H_2_O_2_ activates p90RSK by ROS via Fyn and Ras to activate several transcription factors [51]. The nuclear erythroid 2 p45-related factor-2 (Nrf2), a transcription factor that mediated cytoprotective response against stress, is inhibited by expression of cav-1 [52], leading to alteration of endothelial barrier.

ROS not only induce cav-1 upregulation, but also cause phosphorylation of cav-1 via activation of c-Abl, an upstream kinase of cav-1 [53, 54], to promote the growth of microscopic voids (caveolae formation) within the cellular bilayer [55]. Prdx1 is one of the antioxidant enzymes that plays a protective role in cells against oxidative stress. In cytoplasm, Prdx1 exists as a protein complex with c-Abl-SH domain and protects c-Abl from phosphorylation. Under oxidative stress, oxidant dissociates Prdx1 and c-Abl complex and then induces c-Abl phosphorylation [56], therefore leading to caveolae formation. In addition, Jin and Michel found [57] that actin-binding protein myristoylated alanine-rich C-kinase substrate (MARCKS), an important mediator of the oxidative stress (H_2_O_2_), could induce endothelial permeability change by regulating cytoskeletal reorganization in endothelial cells via a signaling cascade from Rac1 to Ab1, phospholipase C*γ*1, and PKC*δ*, eliciting altered endothelial permeability.

**Figure 1:**
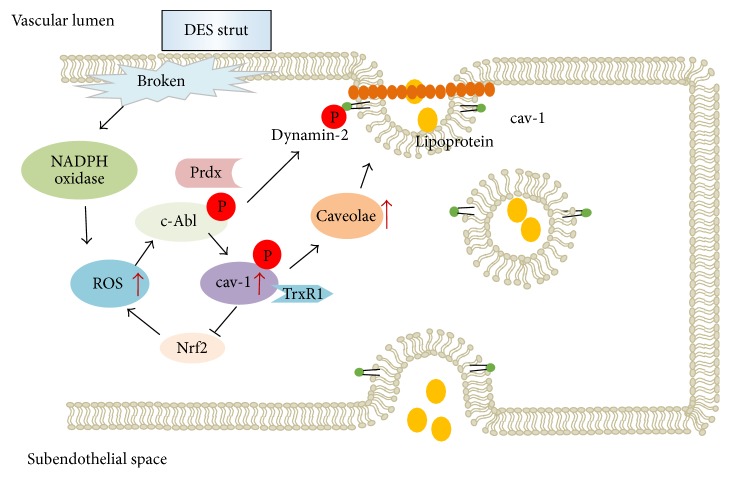
Caveolae-associated transcytosis. Dysfunctional endothelial cells increase lipoprotein uptake by increased cav-1 expression and phosphorylation induced by DES-mediated ROS production.

Fission of caveolae is an important progress in migration of caveolae to the basal membrane for endothelial cells. The GTPase dynamin on oligomerization plays a crucial role in transcytosis for triggering fission by constriction of caveolae necks. Dynamin-2 is mainly involved in the scission of newly formed caveolae from the membrane by self-assembles to form spiral structures on the neck of invaginated pits during endocytosis by stimulating its GTPase activity. Activated* Src* kinase increases dynamin-2 phosphorylation, promotes association with cav-1, and localizes dynamin to caveolae, hence increasing macromolecule transports [58]. Oxidative stress-induced ROS cause recruitment of dynamin 2 and c-Abl to caveolin-enriched microdomains [59]; c-Abl tyrosine-phosphorylated dynamin 2 enhances p47phox/dynamin 2 association, therefore increasing the number and fission of caveolae formation and leading to increased lipid uptake and retention within vessels [59], as shown in Figure 1.

### 4.3. Paracellular Transport Pathway

Under physiological conditions, endothelial cell-cell contacts and vascular endothelial barrier integrity are mediated by tight junctions, adherens junctions, and gap junctions to regulate physiological process, especially inhibiting inflammatory cell transendothelial migration (TEM). Opening of interendothelial cell-cell junctions or disruption of endothelial-matrix contacts within the vasculature after DES placement has a pivotal role in inducing inflammatory cell infiltration, including macrophages, eosinophils, multinuclear giant cells, and lymphocytes [60, 61]. Antiproliferative drugs, potent mTOR inhibitor, may be important in disrupting cell-cell conjunctions [62].

Vascular endothelial- (VE-) cadherin and its associated catenins are important to form adherens junction complexes for controlling endothelial cell-cell adhesion. Data from mTORC2 deficient mice showed that a blocking mTOR or the upstream kinase phosphoinositide 3-kinase (PI3K) dose-dependently decreased VE-cadherin mRNA and protein expression [63]. Furthermore, prolonged rapamycin treatment significantly decreased cytoskeletal adaptor protein Nck by reducing the expression of total mTOR, rictor expression, and mTORC2 formation, finally leading to high permeability [64]. This concept has been confirmed by Walid et al. [65] who clarified that rapamycin, a specific mTOR inhibitor, damaged cell junctions and subsequent tubule formation, facilitating inflammatory cell TEM.

**Figure 2:**
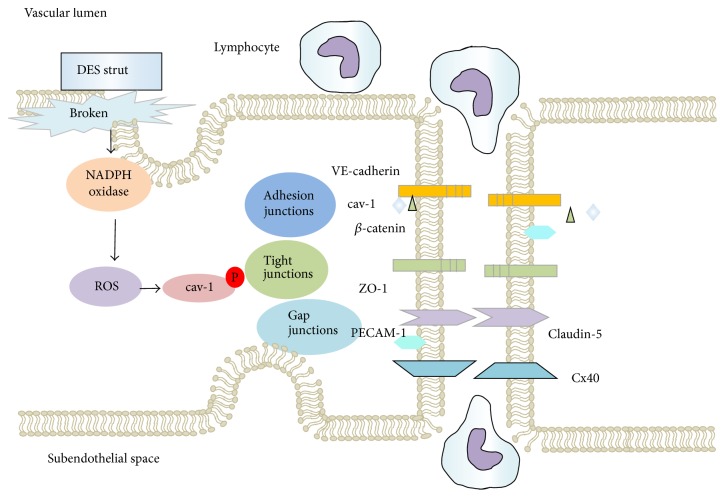
Paracellular-associated pathway. DES-induced dysfunctional endothelial cells show broken cell-cell junctions, further eliciting inflammatory cell migration.

Oxidative stress-induced endothelial cell filamin translocation (from the membrane to the cytosol), cytoskeletal rearrangement, and intercellular gap formation are related to increased monolayer permeability, which contribute to destabilization of junctions [66]. As mentioned above, sirolimus- and paclitaxel-DES induced increase in ROS production may be associated with disruption of endothelial integrity, as shown in Figure 2.

VE-cadherin plays a key role in maintaining cell-cell integrity. The VE-cadherin cytoplasmic tail is highly homologous to other cadherins and binds *β*-catenin or *γ*-catenin. *β*- or *γ*-catenin binds *α*-catenin to stabilize the adherens junction anchorage to the actin cytoskeleton. In addition, confocal images and coimmunoprecipitation technology show significant colocalization of cav-1 and *β*-catenin at cell-cell borders in a nonphosphorylated state [36]. Disruption of binding between *β*-catenin and VE-cadherin interferes with the association of adherens junctions with the actin cytoskeleton, therefore resulting in decreased cell adhesion strength and subsequent barrier disruption [67]. H_2_O_2_ induces the disassociation between cav-1 and *β*-catenin at the endothelial cells borders in cav-1 phosphorylated state. *β*-catenin is tyrosine phosphorylation by ROS via redox-sensitive proline-rich tyrosine kinase 2 [68]. Then, the association of VE-cadherin and *β*-catenin is reduced upon H_2_O_2_ stimulation [69], and *β*-catenin translocates into cytosolic compartment, resulting in endothelial barrier disruption. These results suggest that loss of cell-cell conjunctions is partly induced by disruption of *β*-catenin/VE-cadherin complexes via oxidant-induced paracellular pathway. In the bEnd3 monolayer of mouse endothelial cells, elevated level of cellular ROS led to VE-cadherin and zona occludens-1 (ZO-1) disruption, whereas antioxidant (*N*-acetylcysteine and tempol) treatment significantly lowered the permeability induced by ROS [70].

In addition to decreasing assembly of adherens junctions, ROS also affect the formation of endothelial tight junctions. Under pathological conditions, ROS significantly contribute to blood-brain barrier dysfunction and inflammation in the brain by enhancing cellular migration, paralleling with cytoskeleton rearrangements and redistribution of disappearance of tight junctions proteins claudin-5 and occludin [71]. Production of ROS induced by HIV-transactivator of transcription/cocaine activates Ras/Raf/ERK1/2 pathway contributing to disruption of tight junction protein [72]. Hence, opening of tight junctions after endothelium damage facilitates inflammatory cell migration. Conversely, the NADPH oxidase inhibitor DPI reversed the events, including impaired tight proteins ZO-1 and claudin-5, decreased transendothelial electrical resistance, and significantly increased cytosolic ROS in brain endothelial cells, indicating that ROS play a key role in this process [73].

Immunofluorescent staining for the tight junctional protein occludin and ZO-1 demonstrated that oxidant challenge caused a loss of endothelial tight junction organization. Protein phosphatase 2A (PP2A) interacts with epithelial tight junctions and negatively regulates the integrity of the tight junctions. PP2A-calpha protein and PP2A activity were coimmunoprecipitated with occludin, and this coimmunoprecipitation was rapidly increased by H_2_O_2_. H_2_O_2_-induced dephosphorylation of occludin on threonine residues and redistribution of occludin and ZO-1 from the intercellular junctions is caused by a Src kinase-dependent mechanism [74]. Rhodamine phalloidin staining of the actin cytoskeleton showed that H_2_O_2_ stimulated increased stress fiber formation with concomitant gap formation between adjacent endothelial cells [75]. In addition, ROS could activate myosin light chain kinase, followed by decreased tight junction complex, while providing antioxidant prevented brain endothelial injury [76].

Connexins (Cx) are recognized as structural constituents of gap-junctional intercellular communication (GJIC). Downregulated Cx43 expression and damaged GJIC function in HUVECs along with intracellular ROS production were observed by asymmetric dimethylarginine (ADMA), whereas these events could be attenuated by NADPH oxidase inhibitor [77].

Adhesion molecules that localize at endothelial cells junctions or cell-cell contracts regions are essential to the process of TEM. Activated endothelial cells presented with cell-surface adhesion molecules expressions [78] cause monocytes fixed adhesion on the endothelium, facilitating TEM via paracellular pathway. These molecules include platelet/EC adhesion molecule-1 (PECAM-1), CD99, junctional adhesion molecules A and C (JAM-A and JAM-C), and JAM-like protein (JAML) [79]. Among these adhesion molecules, PECAM-1 acts as a sensor of oxidative stress during the process of TEM. PECAM-1-mediated TEM is dependent on its tyrosine phosphorylation. An inducer of oxidative stress (t-BuooH) in HUVECs caused twofold increase in the TEM of monocyte like HL-60 cells and a fivefold increase in PECAM-1 phosphorylation [80]. H_2_O_2_ supports PECAM-1/SHP-2 complex formation via an “oxidative burst” and sufficiently high concentrations of H_2_O_2_ for a sufficiently long period of time [81], a process that is similar to DES implantation. Conversely, antioxidant enzyme and superoxide dismutase conjugated with antibodies to PECAM-1 quench the corresponding ROS and alleviate vascular oxidative stress and inflammation.

ROS disassemble the endothelial cell actin dense peripheral band, followed by an increase in the number and diameter of intercellular gaps. Millimolar concentrations of reactive oxygen metabolites lead to nonspecific endothelial cell injury, and micromolar concentrations activate inflammatory second messenger cascades which produce distributional changes in endothelial cell cytoskeletal proteins, causing translocation of filamin, attributed to rearrangement of the dense peripheral band of F-actin [82].

## 5. Biodegradable Polymer Stents and Drug-Eluting Absorbable Stents

The slow and often incomplete endothelial regrowth after injury is the primary cause of serious short- and long-term complications, including thrombosis and neoatherosclerosis. Rapid endothelium restoration has the potential to prevent these sequelae [83]. The emergence of biodegradable polymer stents seems attractive because its polymer degrades and eliminates itself from the body leaving the permanent metallic stent without polymer, which would facilitate reendothelialization. Karjalainen et al. [84] performed a clinical study enrolling 44 patients with acute coronary syndrome receiving either a biodegradable polymer-based SES (BP-SES) or durable polymer-based ZES (DP-ZES). Results showed that BP-SES provided better stent strut coverage at 3 months compared with the DP-ZES group, although neither was fully covered. However, a 5-year follow-up research, in which 30 patients with 33 stents (10 with 12 biodegradable polymer biolimus-eluting stents [BES], 10 with 11 SES, and 10 with 10 BMS) showed that lipid-laden neointima with BES had no statistical discrepancy when compared with those with SES or BMS, respectively [85]. Therefore, the safety of biodegradable stents should still be further developed.

Several prospective, multicenter, clinical trials have been performed to directly investigate the effect of bioabsorbable stents on neointimal function. Mattesini et al. [86] designed a clinical study in which 100 complex coronary lesions were treated with a bioabsorbable vascular scaffold (BVS) or second-generation DES. The findings showed that the BVS group had a higher tissue prolapsed area and greater incidence of incomplete strut apposition at the proximal edge compared to the DES group. Furthermore, Christiansen et al. [87] published the results of the SORT OUT V trial in which 2468 patients received either BVS or SES, showing that significantly more patients in the BVS group had definite thrombosis at 12 months than those in the SES group (risk difference, 0.6%; *P* = 0.034), suggesting that the effects of biodegradable stents on vascular reendothelialization merit further validation.

Endothelial dysfunction or damage by oxidants is associated with an enhanced risk of platelet activation and subsequent atherothrombotic complications [88]. To investigate the biocompatibility of biodegradable polymers, cultured monocytes differentiated into functional macrophages were incubated with various polymers including poly-L-lactide, polycaprolactone, or poly-D,L-lactide-co-glycolide for up to 5 days and showed that biodegradable polymers were associated with macrophage adhesion, NADPH oxidase-induced generation of ROS, and excess apoptosis [89]. Furthermore, Hietala et al. [90] examined the possible differences between biodegradable polylactide (PLA) and stainless steel (SS) stents in platelet attachment and morphology after whole blood perfusion. Results revealed that more platelets deposited on PLA stents than on SS stents under all study conditions (*P* < 0.03), while among all biodegradable stents, the braided PLA stent coated with PCL-PLA-heparin accumulated the fewest platelets (*P* < 0.02), indicating that materials, design, and coating techniques of biodegradable stents must be further developed.

ROS could activate platelets, increasing their adhesion to the vascular wall. Evidence showed that platelet recruitment (PR) inhibited by rosuvastatin was associated with downregulation of platelet release of the prothrombotic molecule CD40L, lower production of platelet ROS and isoprostane, and activation of the glycoprotein IIb/IIIa. Detailed mechanisms revealed that platelet isoprostane formation, platelet CD40L, and sNOX2-dp mainly depend on NADPH oxidase, and inhibition of NOX2-derived oxidative stress could impair platelet activation [91]. Moreover, the soluble CD40L (sCD40L)/CD40 axis is a thromboinflammmatory mediator that affects platelet and endothelial functions. Khzam et al. [92] found that pretreatment of early outgrowth cells (EOCs) with sCD40 reduced their inhibitory effect on platelet aggregation. In contrast, blockade of ROS reversed the effects of sCD40-treated EOCs on platelet aggregation. Similar results were observed in disease of anoxia-reoxygenation, where platelets undergoing anoxia-reoxygenation simultaneously increase ROS, thromboxane (Tx) B_2_, and isoprostanes. These events were associated with NOX2 activation and could be inhibited by NOX2-blocking peptide, vitamin C, and the inhibitor of phospholipase A_2_ [93]. Therefore, production of ROS is an important factor in platelet recruitment and thrombotic events, and strategies to decrease oxidative stress can encourage reendothelialization and reduce the incidence of NA.

## 6. Effects of Antioxidants on Reendothelialization after Vascular Injury

Targeting of endothelial function by antioxidants may be promising to promote endothelial healing and to prevent against NA formation after stent implantation. Early trial of probucol (an antioxidant) administration in animal models of stent implantation showed antirestenosis and antithrombotic properties, which are related to promoting in-stent reendothelialization [94]. Moreover, in another study, animals of expanded polytetrafluoroethylene grafts in the abdominal aorta were treated with probucol, showing increased endothelial cell coverage and decreased intimal hyperplasia, suggesting that reducing oxidative stress promotes healing of prosthetic grafts [95]. The mechanisms involved beneficial effects on oxidative stress and improvement of endothelial functional activities and reduced LDL oxidative state [96].

Hanratty et al. [97] found that low flow resulted in greater lumen loss in segments form the same vessel subject to balloon injury, by greater enhancement, whereas the antioxidant pyrrolidine dithiocarbamate effectively reduced intima formation and inward remodeling after balloon-injured vessels. Cicero et al. [98] performed a crossover, double-blind, placebo-controlled randomized clinical trial to detect if a short-term treatment with monacolins combined with antioxidant, red yeast rice, improves lipid pattern and endothelial function in a small cohort-moderately hypercholesterolemic subjuncts. Results showed that monacolin treatment with red yeast rice appeared to safely reduce cholesterolemia, hs-CRP, and improve endothelial function. The detailed mechanisms for improvement of endothelial function are via inhibiting of oxidative stress, downregulating cav-1, upregulating eNOS expression, and decreasing whole blood viscosity [99]. These data suggest that strategies that promote functional healing of vascular endothelium may be cardinal methods, consequently decreasing the risks of late complications [100].

## 7. Conclusion and Perspectives

In summary, it is already demonstrated that reendothelialization with complete function is essential to maintain safety and performance of coronary stents. ROS are released following PCI and are closely associated with neoatherosclerosis formation, which is one of the main mechanisms for late thrombosis. The use of antioxidants may inhibit such complications, by encouraging endothelial coverage, improving endothelial function via mediating lipid uptake and inflammation. Although the current DES and advanced biodegradable stents have tried their best to reduce the rates of adverse cardiac events, including nonfatal myocardial infarction, stroke, repeat target vessel revascularization, or death, inadequate endothelial healing in stented segments plays a key role in the dilemma. It is likely that stents which conquer the overwhelming influence of ROS on the arterial healing process will gain a delighted achievement.

## Figures and Tables

**Table 1:** Different characteristics of NA between BMS and DES.

	Neointima of BMS	Neointima of DES
Tissue components	Smooth muscle cells and proteoglycans-rich extracellular matrix	Fibrin deposition; proteoglycan deposition; larger necrotic; calcified components; and less smooth muscle cells
Lipid-laden plaque	Small	Large
Inflammatory cell infiltration	Less Inflammatory cell infiltration	Many kinds of inflammatory cells: macrophages, multinucleated giant cells, lymphocytes, and granulocytes
Developing time	Slow (>1 year after stenting)	Rapid (<1 year after stenting)
Type of NA	Less of thin-cap NA	Thin-cap NA
Progression	Slow or disappeared	Growing
Prognosis	Relatively stable	High frequency of late stent failure

## Abbreviations

ROS: Reactive oxygen species
BMS: Bare-metal stent(s)
DES: Drug-eluting stent(s)
NA: Neoatherosclerosis
OCT: Optical coherence tomography
PES: Paclitaxel-eluting stent(s)
SES: Sirolimus-eluting stent(s)
IVUS: Intravascular ultrasonography
cav-1: Caveolin-1
eNOS: Endothelial nitric oxide synthase.
